# Development and validation of an interpretable machine learning scoring tool for estimating time to emergency readmissions

**DOI:** 10.1016/j.eclinm.2022.101315

**Published:** 2022-03-06

**Authors:** Feng Xie, Nan Liu, Linxuan Yan, Yilin Ning, Ka Keat Lim, Changlin Gong, Yu Heng Kwan, Andrew Fu Wah Ho, Lian Leng Low, Bibhas Chakraborty, Marcus Eng Hock Ong

**Affiliations:** a Programme in Health Services and Systems Research, Duke-NUS Medical School, 8 College Road, 169857, Singapore; b Health Services Research Centre, Singapore Health Services, Singapore; c Institute of Data Science, National University of Singapore, Singapore; d School of Population Health and Environmental Sciences, Faculty of Life Sciences and Medicine, King's College London, London, United Kingdom; e National Institute for Health Research (NIHR) Biomedical Research Centre, Guy's and St Thomas' NHS Foundation Trust and King's College London, London, United Kingdom; f Department of Internal Medicine, Peking Union Medical College Hospital, Chinese Academy of Medical Sciences and Peking Union Medical College, Beijing, China; g Department of Emergency Medicine, Singapore General Hospital, Singapore; h Department of Family Medicine and Continuing Care, Singapore General Hospital, Singapore; i Department of Post-Acute and Continuing Care, Outram Community Hospital, Singapore; j SingHealth Duke-NUS Family Medicine Academic Clinical Program, Duke-NUS Medical School, Singapore; k Department of Statistics and Data Science, National University of Singapore, Singapore; l Department of Biostatistics and Bioinformatics, Duke University, Durham, NC, United States

**Keywords:** Patient readmission, Emergency, Scoring system, Interpretable machine learning, AutoScore

## Abstract

**Background:**

Emergency readmission poses an additional burden on both patients and healthcare systems. Risk stratification is the first step of transitional care interventions targeted at reducing readmission. To accurately predict the short- and intermediate-term risks of readmission and provide information for further temporal risk stratification, we developed and validated an interpretable machine learning risk scoring system.

**Methods:**

In this retrospective study, all emergency admission episodes from January 1st 2009 to December 31st 2016 at a tertiary hospital in Singapore were assessed. The primary outcome was time to emergency readmission within 90 days post discharge. The Score for Emergency ReAdmission Prediction (SERAP) tool was derived via an interpretable machine learning-based system for time-to-event outcomes. SERAP is six-variable survival score, and takes the number of emergency admissions last year, age, history of malignancy, history of renal diseases, serum creatinine level, and serum albumin level during index admission into consideration.

**Findings:**

A total of 293,589 ED admission episodes were finally included in the whole cohort. Among them, 203,748 episodes were included in the training cohort, 50,937 episodes in the validation cohort, and 38,904 in the testing cohort. Readmission within 90 days was documented in 80,213 (27.3%) episodes, with a median time to emergency readmission of 22 days (Interquartile range: 8-47). For different time points, the readmission rates observed in the whole cohort were 6.7% at 7 days, 10.6% at 14 days, 13.6% at 21 days, 16.4% at 30 days, and 23.0% at 60 days. In the testing cohort, the SERAP achieved an integrated area under the curve of 0.737 (95% confidence interval: 0.730-0.743). For a specific 30-day readmission prediction, SERAP outperformed the LACE index (Length of stay, Acuity of admission, Charlson comorbidity index, and Emergency department visits in past six months) and the HOSPITAL score (Hemoglobin at discharge, discharge from an Oncology service, Sodium level at discharge, Procedure during the index admission, Index Type of admission, number of Admissions during the last 12 months, and Length of stay). Besides 30-day readmission, SERAP can predict readmission rates at any time point during the 90-day period.

**Interpretation:**

Better performance in risk prediction was achieved by the SERAP than other existing scores, and accurate information about time to emergency readmission was generated for further temporal risk stratification and clinical decision-making. In the future, external validation studies are needed to evaluate the SERAP at different settings and assess their real-world performance.

**Funding:**

This study was supported by the Singapore National Medical Research Council under the PULSES Center Grant, and Duke-NUS Medical School.

**Research in context**

**Evidence before this study**

We searched PubMed from 2010 to 2021. Search terms included ("patient readmission" OR "rehospitalization" OR "readmission") AND ("predict" OR "prediction") AND ("emergency department" OR "accident & emergency" OR "emergency" OR "unplanned" OR "avoidable"). We observed that most readmission risk scoring systems were designed to predict 30-day readmission risk, such as the LACE index and the HOSPITAL score, and few scoring systems were able to provide information about time to readmission or quantified change in risk over time.

**Added value of this study**

The new tool, Score for Emergency ReAdmission Prediction (SERAP), is parsimonious and easy to use with only six variables. The SERAP outperforms other existing tools in predicting 30-day readmission risk. Risks for readmission could also be predicted at the 30-day time point and any time point during the 90-day period. This allows for quantifying change in readmission risk over time, significantly increasing real-life practicality.

**Implications of all the available evidence**

This study emphasizes the importance of lab values and personal medical history as predictors of emergency readmission risk, consistent with previous clinical studies. The interpretable machine learning-based tool allows physicians to efficiently calculate patients' readmission risks at various time points and the timing of readmission. Accurate information regarding time to emergency readmission is helpful for further temporal risk stratification, clinical decision-making, and personalized transitional care coordination.

Alt-text: Unlabelled box

## Introduction

Emergency readmission, also known as unplanned readmission, usually refers to patients being readmitted to inpatient service through the emergency department (ED). This poses a challenge, especially for healthcare systems with only finite resources that struggle to meet the rapidly increasing demand brought by the aging population .^1^ In the United States, the emergency readmission rate was estimated to be 18%, incurring an annual cost of $17 billion among Medicare beneficiaries.^2^ Another study in the United Kingdom reported 5.8 million emergency readmission cases during a 6-year period, adding up to an emergency readmission rate of 7%.^3^ In Singapore, a city-state located in Southeast Asia, the all-cause 30-day readmission rate was reported as 11.6% in 2010, and 19.0% for elderly patients.^4^

It has been reported that emergency readmission is associated with multiple factors^5^, and a considerable proportion of readmissions are avoidable.^6^ Hospitals and public health authorities worldwide have been putting forward measures and policies aiming to reduce the rate of readmission. At the policy level, the United States established the Hospital Readmission Reduction Program^7^, where hospitals are financially penalized for high readmission rates. Commonly practiced measures include home-visiting programs, telemonitoring, and patient education upon discharge.^8^

Identifying patients at high risk for emergency readmission is a cornerstone of transitional care interventions that aim to minimize emergency readmission.^9^^,^^10^ Multiple risk scoring systems have been proposed, such as the LACE index (Length of stay, Acuity of admission, Charlson comorbidity index, and Emergency department visits in past six months)^11^ and the HOSPITAL score (Hemoglobin at discharge, discharge from an Oncology service, Sodium level at discharge, Procedure during the index admission, Index Type of admission, number of Admissions during the last 12 months, and Length of stay).^12^ However, these scores have several limitations. Firstly, as both were designed to predict 30-day readmission risk, their performance in predicting longer-term (i.e., > 30 days) risks is not guaranteed ^13^^,^^14^, possibly because the risk of longer-term readmission is determined by different factors.^15^^,^^16^ Therefore, in comparison to patients at short-term (30 days or less) risks, probably different interventional approaches are required to achieve an optimal outcome for those at risk of intermediate-term readmission.^8^ Moreover, in terms of time to readmission or the quantified change in risk over time, little information was elicited from these two scores. This causes some uncertainty in scheduling and coordinating the transitional care program, which may lead to the untimely delivery of interventions and thus reduce the overall effectiveness.^8^ It would be ideal if clinicians knew who should receive transitional care and when interventions were indicated.

To overcome the limitation of current readmission predictive scores, we proposed and validated a scoring system of emergency readmission risk prediction, namely the Score for Emergency ReAdmission Prediction (SERAP), developed through a machine learning-based clinical score generator.^17^ The performance of SERAP was evaluated in a testing cohort and compared with the HOSPITAL score and LACE index.

## Methods

### Study design and setting

This study was a retrospective analysis of patients admitted to Singapore General Hospital (SGH) through ED. Singapore is a city-state in Southeast Asia, with a rapidly aging population^18^; about 1 in 5 Singaporeans are aged 60 or above in 2020.^19^ SGH is the largest public tertiary hospital in Singapore. In the SGH ED, over 120,000 visits were received, and over 36,000 patients were referred for inpatient admissions annually.^20^ In our study, we focus on all index emergency admission episodes and look at their following emergency readmission. Electronic Health Record (EHR) data analyzed in this study were obtained from Singapore Health Services.

### Ethics, consent and permissions

This study was approved by Singapore Health Services' Centralized Institutional Review Board (CIRB 2021/2122), and a waiver of consent was granted for EHR data collection.

### Study population

All index emergency admission episodes^21^ from January 1st, 2009, to December 31st, 2016, were included and followed for 90 days after discharge to determine whether an emergency readmission event occurred. Readmission episodes through non-ED visits were not counted towards the number of emergency readmission. Patients under 21 years old were excluded from our study. Patients deceased during index admission or before possible readmission were also excluded. Non-resident foreign citizens were excluded, as they may not have a complete medical history recorded in our EHR system. Index admission episodes from 2009 to 2015 were randomly split into two non-overlapping cohorts: a training cohort (80%) and a validation cohort (20%). The admission episodes dated in 2016 were assigned to the testing cohort. Sequential testing design was adopted due to better consistency with real-world scenarios and its ability to determine whether our model's performance could be influenced by population shift.

### Outcome and candidate variables

Data were extracted from the hospital's EHR through the SingHealth Electronic Health lntelligence System (eHints), and the data was de-identified in accordance with data governance regulations. The primary outcome was time to emergency readmission within 90 days post discharge. Comorbidities were obtained from hospital diagnoses and discharge records within five years preceding patients' index emergency admissions. All diagnoses were recorded in the form of International Classification of Diseases (ICD) codes (ICD-9/ICD-10) ^22^, which is a globally adopted diagnostic tool for epidemiological and clinical purposes. Comorbidity variables were extracted according to the Charlson Comorbidity Index (CCI).^23^ The algorithms previously proposed by Quan et al.^24^ were applied in this study for the linkage between CCI and ICD codes. A total of 45 preselected candidate variables were collected, according to data availability, clinician's perspectives, and literature reviews. They were all available before discharge from index admission to ensure that the SERAP is capable of early stratification of readmission risks. Candidate variables included demographics, comorbidities, medical utilization history, last measurement of inpatient vital signs and lab tests during the index admission. The list of candidate variables was shown in the supplementary material (eTable 1).

### Statistical analysis

All data were analyzed with R 4.0.2 (R Foundation, Vienna, Austria). For vital signs and lab tests, a certain value would be considered an outlier and marked as missing if it was beyond the plausible physiological range based on domain knowledge, such as any value below zero or a SpO_2_ above 100%. Then, all missing values were imputed with the median value of the training cohort. In the descriptive summaries, baseline characteristics of the dataset were described through univariable and multivariable Cox regression. Kaplan-Meier survival curves were generated for different risk groups stratified by the SERAP and compared through the log-rank test. Furthermore, we computed the 10^th^/25^th^ and 50^th^ percentile readmission times and actual survival probabilities at different time points stratified by our scores.

AutoScore-Survival^17^, a machine learning-based time-to-event score generation algorithm^25^, was implemented to derive the SERAP model. This algorithm combined both machine learning and Cox regression, integrated multiple data manipulation modules, and automated the development of parsimonious sparse-score risk models for time-to-event outcomes. In addition, it builds transparent and straightforward time-to-event scores, which can be easily implemented and validated in clinical practice. In the AutoScore-Survival main flow, tentative SERAP models were generated in the training cohort, and multiple candidate SERAP models were evaluated in the validation cohort for parameter tuning and model selection. Then, the performance metrics of the final SERAP model were calculated based on the testing cohort. The methodology details were shown in the supplementary eMethod.

After model derivation, the predictive performance of the final SERAP model was reported based on the testing cohort, and bootstrapped samples were applied to calculate 95% confidence intervals (CIs). The individual scores were then summed up to derive the aggregate SERAP score for performance evaluation. The predictive power of SERAP was measured by time-dependent area under the curve (AUC(t)) ^26^ concordance indices (Harrell's C-index).^27^^,^^28^ AUC(t) is the most popular and intuitive measure of performance at a specific point of time in time-to-event outcome-predicting models. Meanwhile, C-index is able to summarize the overall performance in a single number, which adapts well to the purpose of this study.^29^ We also derived the integrated AUC (iAUC), a weighted average of AUC(t) ^30^ over a follow-up period (i.e., from Day 1 to Day 90), summarizing the overall discrimination ability of the SERAP. In addition, the SERAP was compared with the LACE index ^11^ and the HOSPITAL score ^12^ within the same testing cohort in terms of predicting time-to-readmission outcomes. Specifically for 30-day readmission prediction, sensitivity, specificity, positive predictive value (PPV), and negative predictive value (NPV) were also computed with the optimal cut-off values, defined as the point nearest to the upper-left corner of the receiver operating characteristic curve.

### Role of the funding source

The funders of the study had no role in study design, data collection, data analysis, data interpretation, or writing of the report. Feng Xie, Nan Liu, Yilin Ning, and Marcus Eng Hock Ong had access to the data. All authors took the decision to submit for publication.

## Results

### Cohort formation and fundamental covariates analysis

**Figure 1:**
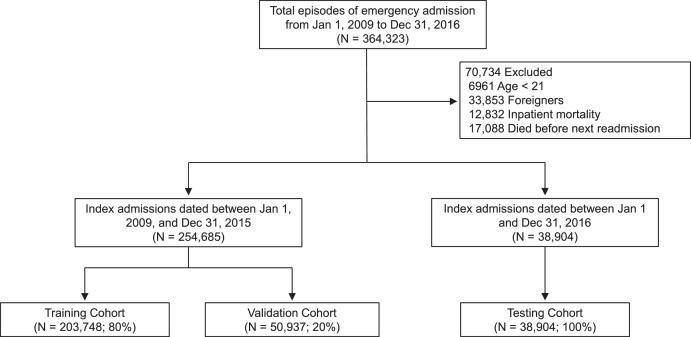
The flow of the study cohort formation

A total of 293,589 ED admission episodes were finally included. As shown in Figure 1, 203,748 episodes were included in the training cohort, 50,937 episodes in the validation cohort, and 38,904 in the testing cohort. The Kaplan-Meier curve of the overall population was plotted in eFigure 1. When censored at the end of the 90-day observation window, 213,376 (72.7%) episodes were readmission-free for more than 90 days. In contrast, 80,213 (27.3%) episodes had readmission within 90 days, with a median time to emergency readmission of 22 days (IQR: 8-47) and a mean time to readmission of 29.5 days (SD=25.3). For different time points, the readmission rates observed in the whole cohort were 6.7% at 7 days, 10.6% at 14 days, 13.6% at 21 days, 16.4% at 30 days, and 23.0% at 60 days. Table 1 summarizes the characteristics of patients with >1 emergency admission episode in the last year, in comparison to those with <=1 emergency admission episode. Table 2 summarizes the univariable and multivariable Cox regression analyses of all prognostic factors. All variables except gender had *P* < 0.001, making it challenging to select a parsimonious model based on *P* values only.

**Table 1:** Baseline characteristics of the whole study cohort.

	Overall	0 or 1 emergency admissions last year	More than 2 emergency admissions last year
# Episodes	293589	234054	59535
Age	63.00 (17.01)	61.94 (17.28)	67.16 (15.22)
Gender			
Female	149741 (51.0%)	120635 (51.5%)	29106 (48.9%)
Male	143848 (49.0%)	113419 (48.5%)	30429 (51.1%)
Race			
Chinese	215803 (73.5%)	173173 (74.0%)	42630 (71.6%)
Others	8543 (2.9%)	6775 (2.9%)	1768 (3.0%)
Indian	30652 (10.4%)	23564 (10.1%)	7088 (11.9%)
Malay	38591 (13.1%)	30542 (13.0%)	8049 (13.5%)
Triage class code			
P3 and P4	78006 (26.6%)	67735 (28.9%)	10271 (17.3%)
P1	49052 (16.7%)	36283 (15.5%)	12769 (21.4%)
P2	166531 (56.7%)	130036 (55.6%)	36495 (61.3%)
Myocardial infarction	17343 (5.9%)	8066 (3.4%)	9277 (15.6%)
Congestive heart failure	35315 (12.0%)	17762 (7.6%)	17553 (29.5%)
Peripheral vascular disease	17896 (6.1%)	8943 (3.8%)	8953 (15.0%)
Stroke	40719 (13.9%)	26334 (11.3%)	14385 (24.2%)
Dementia	8485 (2.9%)	4540 (1.9%)	3945 (6.6%)
Chronic pulmonary disease	31057 (10.6%)	17938 (7.7%)	13119 (22.0%)
Rheumatoid disease	4499 (1.5%)	2830 (1.2%)	1669 (2.8%)
Peptic ulcer disease	12012 (4.1%)	7221 (3.1%)	4791 (8.0%)
Moderate to severe chronic kidney disease	64311 (21.9%)	36057 (15.4%)	28254 (47.5%)
Diabetes			
None	190736 (65.0%)	162946 (69.6%)	27790 (46.7%)
Diabetes without chronic complications	28455 (9.7%)	23008 (9.8%)	5447 (9.1%)
Diabetes with complications	74398 (25.3%)	48100 (20.6%)	26298 (44.2%)
Malignancy			
None	252110 (85.9%)	204520 (87.4%)	47590 (79.9%)
Local tumor, leukemia and lymphoma	24798 (8.4%)	17506 (7.5%)	7292 (12.2%)
Metastatic solid tumor	16681 (5.7%)	12028 (5.1%)	4653 (7.8%)
Number of surgeries	0.29 (0.68)	0.31 (0.69)	0.20 (0.61)
Number of ICU admissions	0.03 (0.27)	0.03 (0.28)	0.02 (0.21)
Length of stay (days)	6.42 (10.89)	6.25 (10.67)	7.09 (11.68)
Time to readmission (days) within 90 days	74.20 (30.42)	79.36 (26.35)	53.94 (36.32)
7-day readmission	19595 (6.7%)	10636 (4.5%)	8959 (15.0%)
14-day readmission	31263 (10.6%)	16867 (7.2%)	14396 (24.2%)
21-day readmission	39895 (13.6%)	21605 (9.2%)	18290 (30.7%)
30-day readmission	48279 (16.4%)	26274 (11.2%)	22005 (37.0%)
60-day readmission	67474 (23.0%)	37449 (16.0%)	30025 (50.4%)
90-day readmission	80213 (27.3%)	45237 (19.3%)	34976 (58.7%)

*Continuous variables are presented as Mean (SD); binary/categorical variables are presented as Count (%).

**Table 2:** Univariable and multivariable Cox regression analysis of the association between included variables and time to emergency readmission within 90 days (N=293,589).

	HR (95% CI)	*p-value*	Adjusted HR (95% CI)	*p-value*
Demographic				
Age	1.019 (1.018-1.019)	<0.001	1.011 (1.010-1.011)	<0.001
Gender				
Female	1[Reference]		1[Reference]	
Male	1.132 (1.116-1.148)	<0.001	1.083 (1.067-1.098)	<0.001
Race				
Chinese	1[Reference]		1[Reference]	
Malay	0.912 (0.893-0.932)	<0.001	0.987 (0.966-1.009)	0.25
Indian	0.967 (0.945-0.99)	0.005	1.009 (0.985-1.033)	0.454
Others	0.883 (0.846-0.922)	<0.001	0.990 (0.949-1.034)	0.664
PACS triage categories				
P3 and P4	1[Reference]		1[Reference]	
P1	2.004 (1.960-2.049)	<0.001	1.400 (1.368-1.433)	<0.001
P2	1.675 (1.645-1.706)	<0.001	1.292 (1.268-1.317)	<0.001
Comorbidities^a^				
Myocardial infarction	2.499 (2.445-2.554)	<0.001	1.064 (1.038-1.090)	<0.001
Congestive heart failure,	2.428 (2.388-2.469)	<0.001	1.236 (1.212-1.26)	<0.001
Peripheral vascular disease	2.295 (2.245-2.346)	<0.001	1.202 (1.173-1.231)	<0.001
Stroke	1.612 (1.584-1.640)	<0.001	1.079 (1.056-1.103)	<0.001
Dementia	1.968 (1.906-2.033)	<0.001	1.176 (1.137-1.217)	<0.001
Chronic pulmonary disease	1.841 (1.807-1.876)	<0.001	1.187 (1.163-1.212)	<0.001
Autoimmune diseases	1.490 (1.421-1.564)	<0.001	1.121 (1.067-1.177)	<0.001
Peptic ulcer disease	1.753 (1.704-1.804)	<0.001	1.078 (1.047-1.11)	<0.001
Hemiplegia or paraplegia	1.645 (1.606-1.686)	<0.001	1.105 (1.073-1.138)	<0.001
Renal diseases	2.460 (2.425-2.496)	<0.001	1.349 (1.322-1.376)	<0.001
Diabetes				
Nil	1[Reference]		1[Reference]	
Diabetes without chronic complications	1.306 (1.275-1.337)	<0.001	1.092 (1.066-1.12)	<0.001
Diabetes with complications	1.926 (1.898-1.955)	<0.001	1.138 (1.119-1.159)	<0.001
Malignancy				
Nil	1[Reference]		1[Reference]	
Non-metastatic solid tumor, leukemia, lymphoma	1.817 (1.779-1.857)	<0.001	1.494 (1.462-1.527)	<0.001
Metastatic solid tumor	2.996 (2.932-3.062)	<0.001	2.786 (2.724-2.849)	<0.001
Liver diseases	1.722 (1.694-1.750)	<0.001	1.198 (1.178-1.219)	<0.001
Health utilization during index admission				
Number of surgeries	0.899 (0.888-0.910)	<0.001	0.928 (0.917-0.94)	<0.001
Number of ICU admissions	1.064 (1.039-1.090)	<0.001	0.937 (0.904-0.971)	<0.001
Number of HDU admission	0.906 (0.892-0.921)	<0.001	0.902 (0.888-0.917)	<0.001
Length of stay	1.005 (1.005-1.006)	<0.001	1.003 (1.003-1.004)	<0.001
Duration of ICU stays	1.014 (1.008-1.020)	<0.001	1.021 (1.011-1.03)	<0.001
Inpatient lab tests (serum level) and vital				
Albumin	0.933 (0.932-0.934)	<0.001	0.964 (0.963-0.966)	<0.001
Bicarbonate	0.981 (0.979-0.984)	<0.001	1.003 (1.000-1.005)	0.019
C reactive protein (Every 10 units)	1.019 (1.017-1.02)	<0.001	1.002 (1.000-1.004)	0.014
Creatine kinase (Every 10 units)	0.998 (0.998-0.999)	<0.001	0.999 (0.999-1.000)	<0.001
Creatine kinase MB (Every 10 units)	1.039 (1.031-1.048)	<0.001	1.026 (1.011-1.041)	<0.001
Creatinine (Every 10 units)	1.012 (1.011-1.012)	<0.001	1.005 (1.005-1.005)	<0.001
Potassium	1.208 (1.187-1.230)	<0.001	0.965 (0.949-0.981)	<0.001
Procalcitonin	1.003 (1.003-1.004)	<0.001	0.999 (0.998-1.000)	0.014
Prothrombin time	1.026 (1.024-1.028)	<0.001	1.012 (1.010-1.015)	<0.001
Sodium	0.948 (0.946-0.950)	<0.001	0.974 (0.972-0.976)	<0.001
Diastolic BP	0.998 (0.997-0.999)	<0.001	1.001 (1.000-1.001)	0.235
Systolic BP	1.003 (1.003-1.004)	<0.001	0.999 (0.999-0.999)	<0.001
Heart rate	1.012 (1.011-1.012)	<0.001	1.006 (1.005-1.006)	<0.001
SpO_2_	0.975 (0.973-0.978)	<0.001	0.999 (0.996-1.003)	0.763
Temperature	0.891 (0.878-0.903)	<0.001	0.914 (0.901-0.927)	<0.001
Previous health utilization				
Emergency admissions in the past month	2.005 (1.990-2.020)	<0.001	1.236 (1.220-1.252)	<0.001
Emergency admissions in the past year	1.155 (1.154-1.157)	<0.001	1.097 (1.094-1.099)	<0.001
HDU admissions in the past month	1.383 (1.339-1.428)	<0.001	0.996 (0.958-1.036)	0.855
HDU admissions in the past year	1.302 (1.291-1.313)	<0.001	1.015(1.003-1.027)	0.012
ICU admissions in the past month	1.493 (1.384-1.610)	<0.001	1.043 (0.954-1.139)	0.353
ICU admissions in the past year	1.268 (1.251-1.285)	<0.001	0.987 (0.966-1.009)	0.235
Surgeries in the past month	1.557 (1.518-1.596)	<0.001	0.959 (0.929-0.990)	0.009
Surgeries in the past year	1.227 (1.222-1.231)	<0.001	1.056 (1.047-1.064)	<0.001
Year of index admission				
2009	1[Reference]		1[Reference]	
2010	1.073 (1.041-1.105)	<0.001	1.004 (0.974-1.034)	0.811
2011	1.100 (1.068-1.132)	<0.001	1.010 (0.980-1.040)	0.517
2012	1.113 (1.081-1.145)	<0.001	1.045 (1.015-1.076)	0.003
2013	1.097 (1.066-1.129)	<0.001	1.062 (1.031-1.094)	<0.001
2014	1.078 (1.047-1.11)	<0.001	1.078 (1.046-1.111)	<0.001
2015	1.071 (1.04-1.102)	<0.001	1.070 (1.038-1.103)	<0.001
2016	1.088 (1.057-1.12)	<0.001	1.090 (1.058-1.124)	<0.001

HR, Hazard Ratio; ED, Emergency Department; BP, Blood Pressure; SpO_2_, Blood Oxygen Saturation; HDU, High Dependency Unit, wards for people who need closer monitoring, more aggressive treatment, and more extensive nursing care than provided in regular service, but slightly less intensive than those given in intensive care. PACS, Patient Acuity Category Scale, the national emergency triage system in Singapore, where P1 patients are the most serious and P4 are non-emergency.

a The reference group consists of patients without the particular disease.

### Parsimony plot and time-to-event scores

The number of variables was determined by the parsimony plot (i.e., model performance vs. complexity) (eFigure 2) based on the validation cohort, and after balancing model performance (i.e., iAUC) with complexity (number of variables, m), six variables were selected. Performance was not markedly improved with more variables added to the time-to-event score.

**Figure 2:**
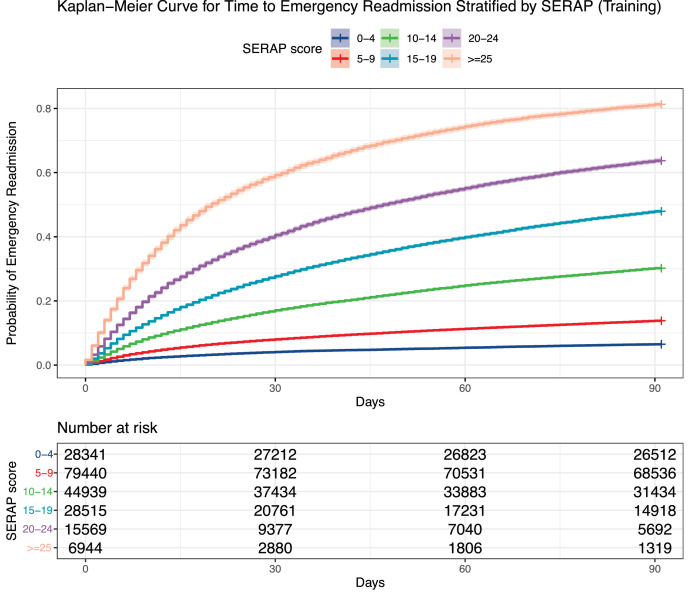

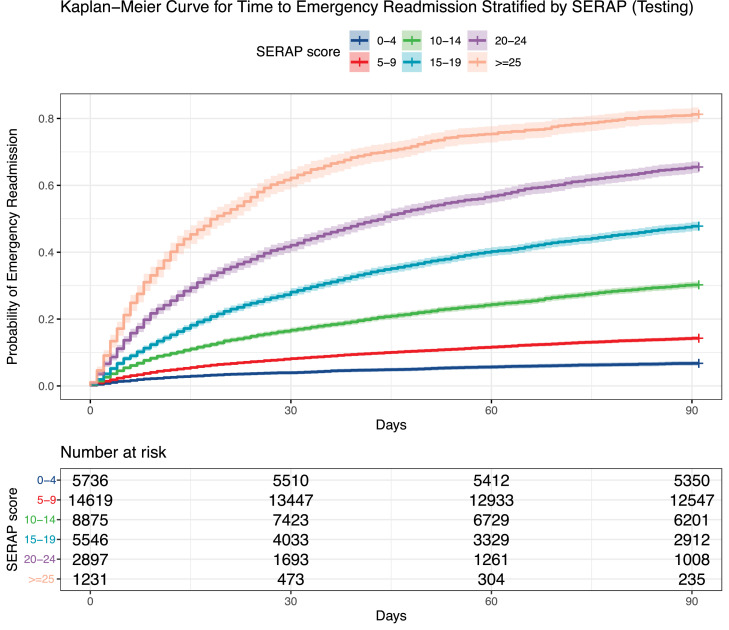
Kaplan-Meier curve of emergency readmission through risk stratification by the SERAP at (a) training cohort and (b) testing cohort

The six-variable survival score, took the number of emergency admissions last year, age, history of malignancy, history of renal diseases, serum creatinine level, and serum albumin level during index admission into consideration, as tabulated in Table 3. The final score ranges from 0 to 40, where a greater number indicates a higher risk of emergency readmission within 90 days after discharge from index admissions. We can find that the number of ED admission last year has the largest score value, accounting for 19 out of 40 points, revealing the most significant contribution to the risk. Table 4 shows different score intervals and their corresponding percentile survival time as well as survival probability estimated by the Kaplan-Meier method. Readmission probability at 7, 14, 30, 60, and 90 days increases with rising SERAP scores, as expected. Table 4 and Figure 2(a) offer a correspondence of scores and predicted readmission probability based on the training set. For example, scores between 15 and 19 correspond to a 30-day readmission probability of 28.1% and a median time to readmission of more than 90 days, while scores ranging from 20 to 24 correspond to a 30-day readmission probability of 42.1% and median time to readmission of shorter than 44 days. As shown in Figure 2(b), the time-to-event score is able to accurately stratify patients in the test set into risk groups based on the Kaplan-Meier curve (*P* < 0.0001).

**Table 3:** Six-variable Score for Emergency ReAdmission Prediction (SERAP).

Variables	Value/Interval	Point
Number of ED admissions last year	0	0
	1	5
	2	7
	3-4	10
	5-6	13
	7-9	15
	≥10	19
Age (years)	<30	0
	30-49	2
	50-79	4
	≥80	5
History of malignancy	Nil	0
	Local tumor, leukemia, and lymphoma	2
	Metastatic	7
History of renal diseases	Nil	0
	Yes	2
Creatinine (umol/L)	<65	1
	65-104	0
	≥105	2
Albumin (g/L)	<24	5
	24-30	3
	31-34	2
	35-39	1
	≥40	0

**Table 4:** SERAP score intervals and their corresponding percentile readmission time or readmission probability at different time points.

Score value range	Percentage of patients	10th percentile readmission time (days)	25th percentile readmission time (days)	Median readmission time (days)	Readmission probability at 7 days (%)	Readmission probability at 14 days (%)	Readmission probability at 30 days (%)	Readmission probability at 60 days (%)	Readmission probability at 90 days (%)
0-4	14.7%	90+	90+	90+	1.9%	2.8%	4.0%	5.7%	6.7%
5-9	37.6%	45	90+	90+	3.4%	5.3%	8.2%	11.6%	14.3%
10-14	22.8%	13	64	90+	6.8%	10.7%	16.7%	24.4%	30.2%
15-19	14.3%	7	25	90+	10.2%	17.2%	28.1%	40.2%	47.8%
20-24	7.4%	4	12	44	17.5%	28.4%	42.1%	56.8%	65.5%
≥25	3.2%	3	7	19	27.5%	43.9%	62.2%	75.4%	81.2%

### Performance evaluation and comparison

The performance of various scoring tools evaluated at different time points in the unseen test set was reported in Table 5. Our SERAP achieved an iAUC of 0.737 (95% CI: 0.730-0.743) and a C-index of 0.744 (95% CI: 0.740-0.748) for time to emergency readmission prediction. eTable 2 shows the sensitivity analysis of SERAP performance by removing each variable, further specifying the impact of previous-year emergency visits. Table 6 specifically compares the performance of predicting 30-day readmission, where SERAP achieved an AUC of 0.753 (95% CI: 0.746-0.759) and outperformed the LACE index (AUC: 0.713, 95% CI: 0.707-0.721) and the HOSPITAL score (AUC: 0.683, 95% CI: 0.676-0.690). In addition, SERAP also had the highest sensitivity of 0.746 (95% CI: 0.735-0.757) and the highest positive likelihood ratio of 1.953 (95% CI: 1.899-2.013) among the three. In addition, SERAP only has six variables, making it easier to implement in clinical practice. Besides 30-day readmission, SERAP could predict readmission rate at any time point during the 90-day period and achieved an AUC of 0.720 (95% CI: 0.713-0.727) for 7-day readmission, 0.737 (95% CI: 0.729-0.745) for 14-day readmission, 0.744 (95% CI: 0.738-0.751) for 21-day readmission, 0.764 (95% CI: 0.760-0.769) for 60-day readmission, and 0.772 (95% CI: 0.767-0.777) for 90-day readmission. Besides, eTable 3 and eTable 4 summarize the comparison of performance in subpopulations stratified by age and gender, respectively. It shows that SERAP may have reduced performance in older patients (age>=60), but is still significantly superior to the other two baseline scores.

**Table 5:** Performance of different scoring systems on the testing cohort.

	SERAP	LACE	HOSPITAL
iAUC	0.737 (0.730-0.743)	0.707 (0.700-0.712)	0.672 (0.665-0.679)
C-index	0.744 (0.740-0.748)	0.714 (0.709-0.718)	0.695 (0.690-0.700)
AUC(t=7)	0.720 (0.713-0.727)	0.695 (0.685-0.705)	0.658 (0.646-0.670)
AUC(t=14)	0.737 (0.729-0.745)	0.706 (0.698-0.712)	0.676 (0.667-0.686)
AUC(t=21)	0.744 (0.738-0.751)	0.710 (0.704-0.716)	0.679 (0.671-0.686)
AUC(t=60)	0.764 (0.760-0.769)	0.724 (0.720-0.730)	0.689 (0.683-0.694)
AUC(t=90)	0.772 (0.767-0.777)	0.730 (0.725-0.735)	0.689 (0.683-0.694)

AUC: area under the curve.

iAUC: integrated area under the curve.

**Table 6:** Comparison of SERAP with LACE and HOSPITAL for predicting 30-day readmission on the testing cohort.

	SERAP	LACE	HOSPITAL
AUC(t=30)	0.753 (0.746-0.759)	0.713 (0.707-0.721)	0.683 (0.676-0.690)
Number of Variables	6	17+3*	4
Cut-off	11	10	4
Sensitivity	0.746 (0.735-0.757)	0.703 (0.691-0.714)	0.685 (0.674-0.696)
Specificity	0.618 (0.613-0.624)	0.618 (0.613-0.624)	0.595 (0.590-0.600)
PPV	0.280 (0.275-0.283)	0.268 (0.264-0.272)	0.252 (0.248-0.256)
NPV	0.924 (0.921-0.927)	0.913 (0.910-0.916)	0.905 (0.901-0.908)
Positive LR	1.953 (1.899-2.013)	1.840 (1.786-1.899)	1.691 (1.644-1.740)
Negative LR	0.432 (0.389-0.481)	0.504 (0.458-0.529)	0.529 (0.507-0.553)

⁎ LACE index consists of the Charlson Comorbidity Index, which contains 17 breakdowns of various comorbidities. AUC: area under the curve PPV: positive predictive value NPV: negative predictive value LR: likelihood ratios.

## Discussion

In this cohort study, a parsimonious and point-based SERAP score was developed for patient stratification according to their readmission risk. The SERAP was validated in a testing cohort and has shown better discriminative power than the LACE index and the HOSPITAL score (AUC 0.753 vs. 0.713 vs. 0.683 based on 30-day emergency readmission). In addition, the SERAP predicted readmission rate at any time point during the 90-day period and achieved a good performance, significantly improving real-life practicality. It is a transparent and interpretable tool with only six variables, making it easy to implement in hospital settings. With this tool, a physician could better understand patients' readmission risks at various time points and when patients might be readmitted.

Some notable findings were also revealed in this study. The number of ED admissions the year before was identified as an essential predicting factor. Frequent admitters were found in our study, with some even exceeding ten admissions in the preceding year ^31^, bringing in a heavy financial burden to society.^32^ Renal diseases, malignancy, and serum albumin level were also identified as key factors for readmission, consistent with previous studies.^33^^,^^34^ Interestingly, in contrast to previous studies in which readmission risk increases with high serum creatine value only^35^^,^^36^, our results suggested that a low level of creatinine also leads to the rise of readmission risk, probably because low creatinine, serving as an indicator for low muscle mass, is associated with other medical conditions, such as diabetes mellitus, chronic liver disease, and malnutrition,^37^^,^^38^ and therefore, contributes to increased mortality and morbidity. This finding calls for more research to comprehensively evaluate the association between serum creatine level and patient outcome. Although the collinearity issue may exist in our scoring system, the model is still valid with the aim of improving the overall predictive performance. In our cohort, the 30-day readmission rate is 16.4%, which is slightly higher compared with a large-scale national study in the US^39^, where the 30-day readmission rate for patient's emergent index admission is 12.7%, mainly due to the rapid-aging population in Singapore.

Several possible reasons why the SERAP outperformed the LACE index and the HOSPITAL score in this study were proposed. To start with, age was not included in both scores as a predictor. The role of patients' age in readmissions has been demonstrated in other studies^40^ ^41^, and more attention should be paid to this factor in a consistently aging population as in Singapore.^42^ In ^43^, the LACE index was shown as a poor tool for predicting readmission in older UK inpatients. Another large-scale US-based study^39^ also affirmed the contribution of age to readmission risk. In addition, the LACE index includes the CCI index, which was developed only based on a longitudinal study of 559 patients in 1987 ^41^, and its weighting strategy may need updating now that the world population is evolving towards a highly aging society with a rising readmission rate.^44^ In comparison, only two vital comorbidities were selected and weighted in our SERAP, based on the readmission risks in a training cohort of 200,000 episodes, making it more practical and more accurate. In contrast, the HOSPITAL score did not include any comorbidities as a predictor, leading to a relatively poor performance in a cohort from an aging population.

In addition to its accuracy, the SERAP has several other strengths. The SERAP is developed and validated based on a time-to-event outcome, where the readmission window ranges from 14 days to 90 days. This endows robust prediction power at both short-term and intermediate-term readmission risks. Such a flexible prediction window increases its usability in comparison to the models designed for 30-day readmission risk only, as many patients continue to receive care beyond 30 days. Thus, the Kaplan–Meier estimator and curve based on the SERAP provide physicians with an exact predicted readmission probability and interpret how it changes over time in an intuitive manner. Moreover, machine learning-based variable selection by AutoScore-Survival can potentially filter out redundant information to achieve a parsimonious solution with only six variables, making it easy to use in real-world clinical settings. In comparison, the LACE index includes the CCI index, requiring information about 17 conditions, which may hinder its implementation in real clinical settings, especially for undeveloped regions where the modern EHR system was not employed.

There are several potentially applicable scenarios for the SERAP in clinical practice. First, it can be used as a risk stratification tool in transitional care planning and coordination. The identification of patients at risk, coordination of care, and the timeliness of follow-ups have been identified as critical factors for successful transitional care ^45^^,^.^46^ Another study suggested that a data-driven approach for scheduling post-discharge interventions leads to a significant reduction in readmission rate by 44.7%, compared to regular visit-based home care.^47^ With the accurate risk stratification in addition to the time-to-readmission outcome prediction, it is more likely to identify the most vulnerable period of each patient, such as the segment of the time-to-readmission risk curve where the slope changes drastically or when the risk exceeds a pre-set threshold. Thus, the scheduling of follow-up can be better tailored and individualized, and timeliness could be ensured. To assist in a readmission reduction program that aims to deliver transitional care before the cumulative readmission risk of a specific patients group reaches 25%, a timeline could be easily drafted with the Kaplan-Meier curve derived from the testing cohort: Patients with SERAP scores equal or greater than 25 should be prioritized to receive intervention before post-discharge day 7, followed by patients with scores within the range of 20∼24, before day 12, and those with scores of 15∼19, before day 25. Furthermore, the SERAP could also be adopted in the practice of reverse triage, a strategy coping with unusually high-demand situations by identifying patients who no longer need in-hospital resources and is safe for early discharge.^48^, ^49^, ^50^ The integration of high-quality readmission risk evaluation models into the decision-making algorithm for reverse triage has been shown to further improve patient safety by Caramello et al.^50^ Further research needs to be conducted to validate the effectiveness and physician-perceived acceptability after incorporating the SERAP into the transitional care model and reverse triage.

The study also has several limitations. First, the dataset was based on the hospital's EHR portal, and it only contains certain system-collected information and does not include all information available that should, in theory, be elicited. Some potential risk factors, such as full blood count, troponin, chief complaint, and psychiatric variables, are not recorded in the system, while manual extraction was not feasible because of the enormous amount of data. Furthermore, our index admission episodes were limited to inpatient admissions through ED due to technical limitations, which might impact the generalizability of all hospital admissions. Future research should look at other types of admission, such as elective admission. Second, this study is based on the data of within the aforementioned eight-year period, where the coronavirus disease 2019 (COVID-19) pandemic was not involved. Future research should further extract recent year data to examine whether the COVID-19 pandemic would influence the performance of the SERAP model. Third, although only around 1% of vital sign data are missing, the missing rate of laboratory tests is higher, reaching more than 20% in some items. Therefore, median value imputation for raw EHR data might not be perfect, which is based on the hypothesis that patients with missing lab tests are more likely to have a result value within a normal range. It hints on the possibility that these lab tests were not performed due to the lack of clinical relevance, and we believe such hypothesis is reasonable, yet it does not hold true in all settings. Last, this was a single-center study at a large teaching hospital, and thus, our findings may not be easily generalized to other settings. The performance of the SERAP may also vary in different healthcare settings. In the future, external validation studies are needed to validate the SERAP at different settings and assess its real-world performance. Prospective data collection is supposed to explore the clinical value and effect of our model in practice and further validate its efficacy.

In conclusion, better performance in emergency readmission risk prediction was achieved by the SERAP than other existing scores, and accurate information about time to emergency readmission was generated for further temporal risk stratification and clinical decision-making.

## Declaration of interests

Feng Xie declares that this study was supported by the Singapore National Medical Research Council under the PULSES Center Grant. Nan Liu declares that this study was supported by Duke-NUS Medical School. All other authors declare that they have no competing interests.

## Appendix Supplementary materials

Image, application 1
