# Supplementary material for: Development and validation of an interpretable machine learning scoring tool for estimating time to emergency readmissions
Source: eClinicalMedicine. 2022 Mar 6;45:101315. doi: 10.1016/j.eclinm.2022.101315 (PMC8904223; doi:10.1016/j.eclinm.2022.101315)
Supplement: Supplementary file 1 [file mmc1.docx]

**Supplementary Material**

eTable 1. List of candidate variables and their definitions.

eTable 2. Sensitivity analysis of SERAP performance by removing each variable.

eTable 3. Comparison of performance achieved by different risk scores on the testing set stratified by age.

eTable 4. Comparison of performance achieved by different risk scores on the testing set stratified by gender.

eFigure 1. Kaplan Meier Curve for time to readmission for the whole cohort.

eFigure 2. Parsimony plot of the number of variables versus integrated area under the curve values for SERAP on the validation cohort.

eMethod. Description of the AutoScore-Survival method.

**eTable 1 List of candidate variables and their definitions.**

| Candidate Variable Name | Definition |
| --- | --- |
| Demographics | |
| Age | Age in years, specific to the index emergency admission |
| Gender | Gender identity as identified in official patient identification documents |
| Race | Chinese, Malay, Indian or others as identified in official patient identification documents |
| Health utilization during index admission | |
| Number of surgeries | Total number of in-hospital surgeries during the index emergency admission period |
| Number of ICU admissions | Total number of Intensive Care Unit admissions during the index emergency admission period |
| Number of HDU admission | Total number of High Dependency Unit admissions during the index emergency admission period |
| Length of stay | Length of stay of the index emergency admission. Unit: days. |
| Duration of ICU stays | The total duration of the total Intensive Care Unit stay during the index emergency admission period. Unit: days. |
| Variable extracted from index admission | |
| Triage class | Triage class obtained when patients visit ED at the index admission. It's based on the national Patient Acuity Category Scale (PACS): (1) PAC1 patients are the most serious, time-critical patients who require immediate attention or resuscitation; (2) PAC2 patients are non-ambulant patients who appear to be in a stable state with no immediate danger of collapse; (3) PAC3 refers to ambulant patients; and (4) PAC4 are non-emergency patients. |
| Albumin | The last measurement of albumin during the index emergency admission period (Unit: g/L) |
| Bicarbonate | The last measurement of bicarbonate during the index emergency admission period (Unit: mmol/L) |
| C reactive protein | The last measurement of C reactive protein during the index emergency admission period (Unit: mg/L) |
| Creatine kinase | The last measurement of creatine kinase during the index emergency admission period (Unit: U/L) |
| Creatine kinase MB | The last measurement of creatine kinase myocardial band during the index emergency admission period (Unit: Ug/L) |
| Creatinine | The last measurement of creatinine during the index emergency admission period (Unit: umol/L) |
| Potassium | The last measurement of potassium during the index emergency admission period (Unit: mmol/L) |
| Procalcitonin | The last measurement of procalcitonin during the index emergency admission period (Unit: Ug/L) |
| Prothrombin time | The last measurement of prothrombin time during the index emergency admission period (Unit: sec) |
| Sodium | The last measurement of sodium during the index emergency admission period (Unit: mmol) |
| Diastolic BP | The last measurement of the diastolic blood pressure (BP) during the index emergency admission period. Typically, electronically obtained during automated blood pressure cuff measurement. (Unit: mmHg) |
| Systolic BP | The last measurement of the systolic blood pressure (BP) during the index emergency admission period. Typically, electronically obtained during automated blood pressure cuff measurement. (Unit: mmHg) |
| Heart Rate | The last measurement of the number of heart beats per minute during the index admission period. Typically, electronically obtained during automated blood pressure cuff measurement. |
| SpO2 | The last measurement of the peripheral capillary oxygen percentage saturation during the index emergency admission period. Typically, electronically obtained using a portable fingertip pulse oximeter. |
| Temperature | The last measurement of the temperature during the index emergency admission period. (Unit: Celsius) |
| Comorbidities (ICD-9 and ICD-10 codes were extracted from patient medical records in the preceding five years and matched to their corresponding comorbidities based on prior work by Quan et. al. Comorbidity was deemed to be absent if the representative codes were not identified) | |
| Myocardial infarction | ICD-9-CM: 410.x, 412.x, or ICD-10: I21.x, I22.x, I25.2 |
| Congestive heart failure | ICD-9-CM: 428.x, or ICD-10: I09.9, I11.0, I13.0, I13.2, I25.5, I42.0, I42.5–I42.9, I43.x, I50.x, P29.0 |
| Peripheral vascular diseases | ICD-9-CM: 443.9, 441.x, 785.4, V43.4, or Procedure 38.48, or ICD-10: I70.x, I71.x, I73.1, I73.8, I73.9, I77.1, I79.0, I79.2, K55.1, K55.8, K55.9, Z95.8, Z95.9 |
| Stroke | E.g. cerebrovascular accident (CVA) or transient ischemic attack (TIA)  ICD-9-CM: 430.x–438.x, or ICD-10: G45.x, G46.x, H34.0, I60.x–I69.x |
| Dementia | ICD-9-CM: 490.x–505.x, 506.4, or ICD-10: F00.x–F03.x, F05.1, G30.x, G31.1 |
| Chronic pulmonary diseases | ICD-9-CM: 290.x, or ICD-10: I27.8, I27.9, J40.x–J47.x, J60.x–J67.x, J68.4, J70.1, J70.3 |
| Autoimmune diseases | ICD-9-CM: 710.0, 710.1, 710.4,714.0–714.2, 714.81, 725.x, or ICD-10: M05.x, M06.x, M31.5, M32.x–M34.x, M35.1, M35.3, M36.0 |
| Peptic ulcer disease | ICD-9-CM: 531.x–534.x, or ICD-10: K25.x–K28.x |
| Diabetes | Diabetes without chronic complications: ICD-9-CM: 250.0–250.3, 250.7, or ICD-10: E10.0, E10.1, E10.6, E10.8, E10.9, E11.0, E11.1, E11.6, E11.8, E11.9,  E12.0, E12.1, E12.6, E12.8, E12.9, E13.0, E13.1, E13.6, E13.8, E13.9, E14.0, E14.1, E14.6, E14.8, E14.9  Diabetes with complications: ICD-9-CM: 250.4–250.6, or ICD-10: E10.2–E10.5, E10.7, E11.2–E11.5, E11.7, E12.2–E12.5, E12.7, E13.2–E13.5, E13.7, E14.2–E14.5, E14.7 |
| Hemiplegia or paraplegia | ICD-9-CM: 344.1, 342.x, or ICD-10: G04.1, G11.4, G80.1, G80.2, G81.x, G82.x, G83.0–G83.4, G83.9 |
| Renal diseases | ICD-9-CM: 582.x, 583–583.7, 585.x, 586.x, 588.x, or ICD-10: I12.0, I13.1, N03.2–N03.7, N05.2–N05.7, N18.x, N19.x, N25.0, Z49.0–Z49.2, Z94.0, Z99.2 |
| Malignancy | Local tumor, leukemia, or lymphoma (except malignant neoplasm of skin): ICD-9-CM: 140.x–172.x, 174.x.–195.8, 200.x–208.x, or ICD-10: C00.x–C26.x, C30.x–C34.x, C37.x–C41.x, C43.x, C45.x–C58.x, C60.x–C76.x, C81.x–C85.x, C88.x, C90.x–C97.x  Metastatic solid tumor: ICD-9-CM:196.x–199.1, or ICD-10:: C77.x–C80.x |
| Liver diseases | Mild liver disease: ICD-9-CM: 571.2, 571.4–571.6, or ICD-10: B18.x, K70.0–K70.3, K70.9, K71.3–K71.5, K71.7, K73.x, K74.x, K76.0, K76.2–K76.4, K76.8, K76.9, Z94.4  Severe liver disease: ICD-9-CM: 456.0–456.21, 572.2–572.8, or ICD-10: I85.0, I85.9, I86.4, I98.2, K70.4, K71.1, K72.1, K72.9, K76.5, K76.6, K76.7 |
| Previous Health utilization | |
| Emergency admissions in the past month | Total number of unique emergency admissions within a time period of one month from the index admission |
| Emergency admissions in the past year | Total number of unique emergency admissions within a time period of one year from the index admission |
| HDU admissions in the past month | Total number of unique High Dependency Unit admissions within a time period of one month from the index admission. HDU represents High Dependency Units, also called step-down, progressive, and intermediate care units. HDUs are wards for people who need closer monitoring, more aggressive treatment, and more extensive nursing care than provided in regular service, but slightly less intensive than that given in intensive care. The ratio of nurses to patients may be slightly lower than in intensive care but higher than in most general wards. |
| HDU admissions in the past year | Total number of unique High Dependency Unit admissions within a time period of one year from the index admission |
| ICU admissions in the past month | Total number of unique Intensive Care Unit admissions within a time period of one month from the index admission |
| ICU admissions in the past year | Total number of unique Intensive Care Unit admissions within a time period of one year from the index admission |
| Surgeries in the past month | Total number of unique surgeries within a time period of one month from the index admission |
| Surgeries in the past year | Total number of unique surgeries within a time period of one year from the index admission |

ICD-9-CM: International Classification of Diseases, Ninth Revision, Clinical Modification. ICD-10: The International Statistical Classification of Diseases and Related Health Problems 10th Revision

**eTable 2.** Sensitivity analysis of SERAP performance by removing each variable.

|  | Removing one variable based on SERAP | | | | | | Original SERAP |
| --- | --- | --- | --- | --- | --- | --- | --- |
|  | Number of ED admissions last year | Age | History of malignancy | History of renal diseases | Creatinine | Albumin |  |
| iAUC | 0.650 (0.642-0.656) | 0.729 (0.721-0.736) | 0.730 (0.724-0.737) | 0.734 (0.727-0.739) | 0.735 (0.730-0.742) | 0.735 (0.729-0.742) | 0.737 (0.730-0.744) |
| C-index | 0.669 (0.665-0.675) | 0.740 (0.735-0.745) | 0.734 (0.729-0.739) | 0.742 (0.738-0.747) | 0.743 (0.739-0.747) | 0.743 (0.740-0.748) | 0.743 (0.739-0.747) |
| AUC (t=7) | 0.636 (0.624-0.646) | 0.712 (0.700-0.724) | 0.717 (0.707-0.725) | 0.717 (0.705-0.726) | 0.717 (0.708-0.726) | 0.716 (0.706-0.727) | 0.719 (0.709-0.729) |
| AUC (t=14) | 0.647 (0.637-0.655) | 0.730 (0.723-0.739) | 0.731 (0.725-0.739) | 0.735 (0.729-0.741) | 0.735 (0.729-0.742) | 0.734 (0.727-0.741) | 0.737 (0.729-0.744) |
| AUC (t=21) | 0.655 (0.647-0.662) | 0.738 (0.732-0.745) | 0.736 (0.728-0.743) | 0.741 (0.736-0.747) | 0.742 (0.736-0.749) | 0.741 (0.736-0.748) | 0.744 (0.737-0.751) |
| AUC (t=30) | 0.664 (0.656-0.671) | 0.745 (0.739-0.752) | 0.741 (0.734-0.748) | 0.750 (0.745-0.755) | 0.751 (0.746-0.757) | 0.749 (0.744-0.754) | 0.752 (0.747-0.758) |
| AUC (t=60) | 0.675 (0.669-0.681) | 0.756 (0.751-0.762) | 0.751 (0.745-0.757) | 0.761 (0.755-0.766) | 0.762 (0.758-0.767) | 0.762 (0.757-0.766) | 0.764 (0.759-0.769) |
| AUC (t=90) | 0.683 (0.677-0.688) | 0.764 (0.758-0.769) | 0.759 (0.753-0.763) | 0.768 (0.763-0.774) | 0.769 (0.765-0.774) | 0.769 (0.765-0.774) | 0.771 (0.766-0.776) |

**eTable 3.** Comparison of performance achieved by different risk scores on the testing set stratified by age .

|  | Subpopulation (Age>=60) | | | Subpopulation (Age<60) | | |
| --- | --- | --- | --- | --- | --- | --- |
|  | SERAP | LACE | HOSPITAL | SERAP | LACE | HOSPITAL |
| iAUC | 0.709 (0.699-0.720) | 0.678 (0.671-0.687) | 0.650 (0.641-0.659) | 0.763 (0.751-0.778) | 0.735 (0.720-0.748) | 0.705 (0.689-0.721) |
| C-index | 0.712 (0.706-0.718) | 0.680 (0.674-0.687) | 0.668 (0.662-0.674) | 0.783 (0.776-0.793) | 0.755 (0.745-0.765) | 0.739 (0.730-0.751) |
| AUC (t=7) | 0.695 (0.683-0.706) | 0.673 (0.663-0.685) | 0.638 (0.625-0.649) | 0.740 (0.721-0.767) | 0.713 (0.695-0.732) | 0.688 (0.665-0.712) |
| AUC (t=14) | 0.711 (0.699-0.722) | 0.679 (0.670-0.689) | 0.654 (0.643-0.663) | 0.760 (0.746-0.776) | 0.731 (0.713-0.744) | 0.709 (0.691-0.729) |
| AUC (t=21) | 0.716 (0.706-0.725) | 0.681 (0.672-0.689) | 0.654 (0.644-0.664) | 0.774 (0.761-0.790) | 0.743 (0.727-0.756) | 0.716 (0.701-0.732) |
| AUC (t=30) | 0.723 (0.714-0.731) | 0.685 (0.677-0.693) | 0.661 (0.653-0.669) | 0.785 (0.773-0.798) | 0.749 (0.734-0.762) | 0.719 (0.705-0.733) |
| AUC (t=60) | 0.734 (0.726-0.742) | 0.691 (0.683-0.698) | 0.665 (0.659-0.672) | 0.796 (0.788-0.808) | 0.763 (0.752-0.775) | 0.724 (0.712-0.736) |
| AUC (t=90) | 0.741 (0.735-0.748) | 0.697 (0.691-0.704) | 0.667 (0.661-0.673) | 0.801 (0.794-0.812) | 0.766 (0.755-0.776) | 0.719 (0.709-0.731) |

**eTable 4**. Comparison of performance achieved by different risk scores on the testing set stratified by gender.

|  | Subpopulation (Male) | | | Subpopulation (Female) | | |
| --- | --- | --- | --- | --- | --- | --- |
|  | SERAP | LACE | HOSPITAL | SERAP | LACE | HOSPITAL |
| iAUC | 0.740 (0.729-0.748) | 0.704 (0.695-0.714) | 0.675 (0.666-0.685) | 0.734 (0.723-0.744) | 0.705 (0.695-0.716) | 0.667 (0.656-0.678) |
| C-index | 0.746 (0.74-0.752) | 0.708 (0.702-0.715) | 0.696 (0.689-0.703) | 0.740 (0.733-0.746) | 0.716 (0.708-0.723) | 0.694 (0.685-0.702) |
| AUC (t=7) | 0.720 (0.706-0.730) | 0.693 (0.681-0.706) | 0.662 (0.650-0.677) | 0.719 (0.703-0.734) | 0.693 (0.679-0.709) | 0.652 (0.634-0.669) |
| AUC (t=14) | 0.741 (0.731-0.751) | 0.702 (0.691-0.712) | 0.678 (0.666-0.690) | 0.733 (0.721-0.746) | 0.704 (0.692-0.717) | 0.672 (0.659-0.687) |
| AUC (t=21) | 0.747 (0.739-0.755) | 0.706 (0.698-0.717) | 0.680 (0.671-0.691) | 0.741 (0.730-0.751) | 0.710 (0.699-0.721) | 0.676 (0.664-0.688) |
| AUC (t=30) | 0.757 (0.750-0.765) | 0.711 (0.702-0.720) | 0.686 (0.677-0.696) | 0.747 (0.737-0.756) | 0.715 (0.705-0.725) | 0.680 (0.669-0.691) |
| AUC (t=60) | 0.769 (0.762-0.775) | 0.718 (0.710-0.727) | 0.693 (0.685-0.701) | 0.759 (0.751-0.767) | 0.727 (0.718-0.736) | 0.683 (0.673-0.691) |
| AUC (t=90) | 0.777 (0.771-0.784) | 0.727 (0.720-0.733) | 0.692 (0.684-0.700) | 0.765 (0.757-0.772) | 0.731 (0.723-0.737) | 0.684 (0.676-0.693) |

**eFigure 1**. Kaplan-Meier Curve for time to readmission for the whole cohort


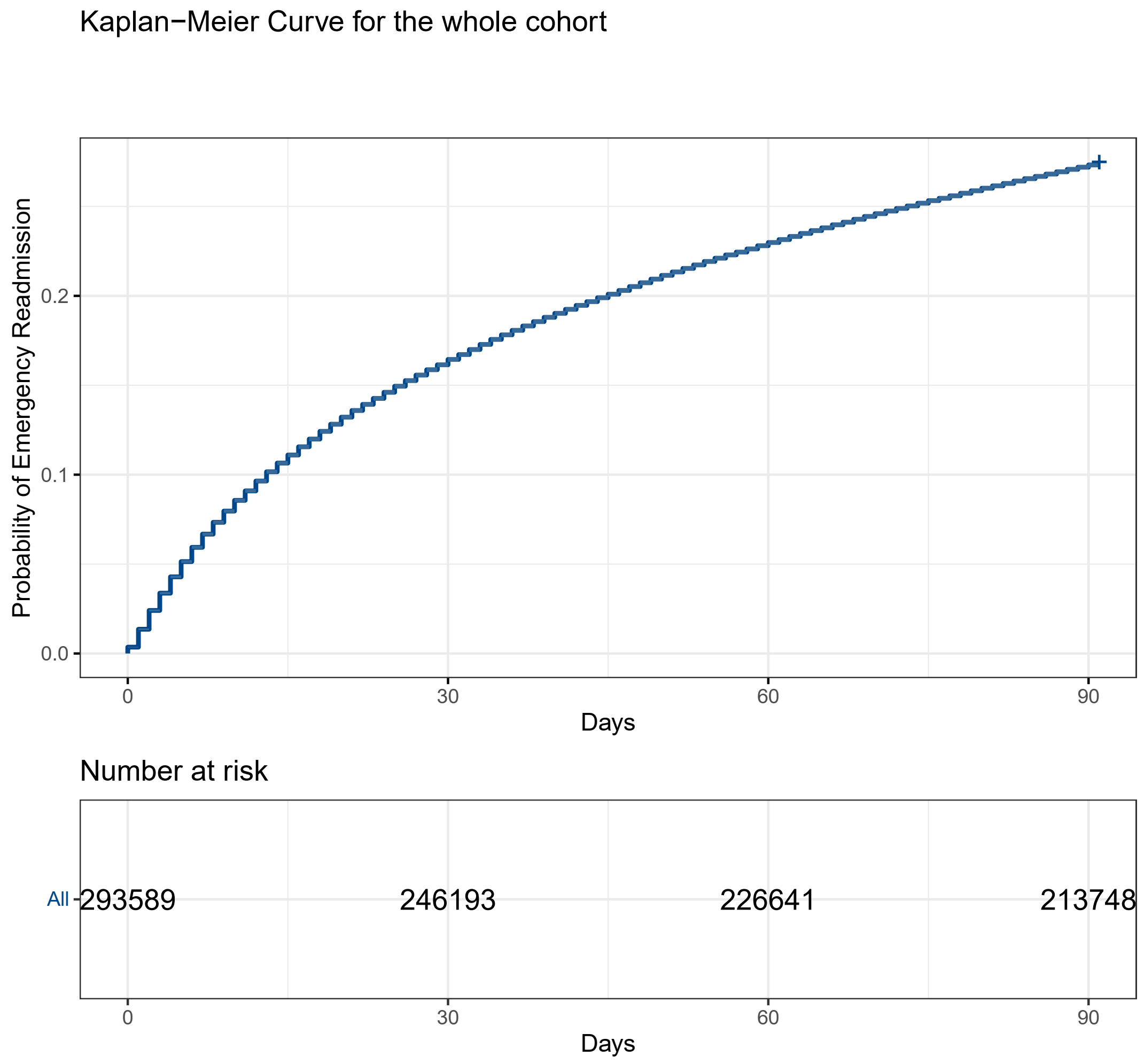
 **eFigure 2**. Parsimony plot of the number of variables versus integrated area under the curve values for SERAP on the validation cohort. The solid black dot shows the selected point for achieving the model parsimony (i.e., number of variables *m* = 6)


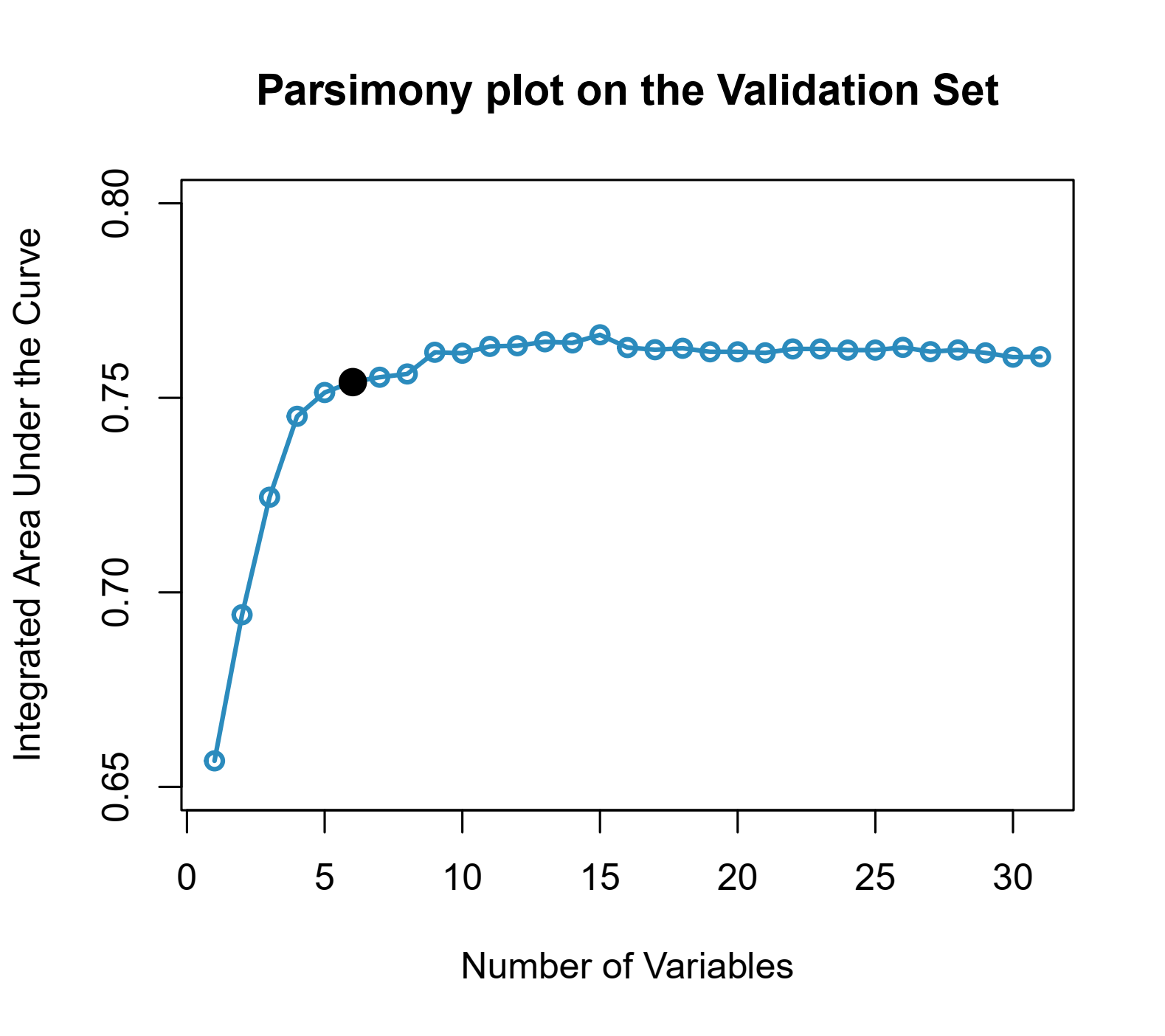


**eMethod.** Description of the AutoScore-Survival method.

Clinical scores have been conventionally derived in 2 ways: through expert opinions or consensus and traditional cohort studies. However, both approaches are labor-intensive and are not easy to update over time. AutoScore was then developed as a genetic method for deriving a parsimonious scoring system with easy access to validation in the context of EHRs. In order to derive a parsimonious time-to-event score, AutoScore was further extended to AutoScore-Survival, with the ability to automate the development of a parsimonious time-to-event score.


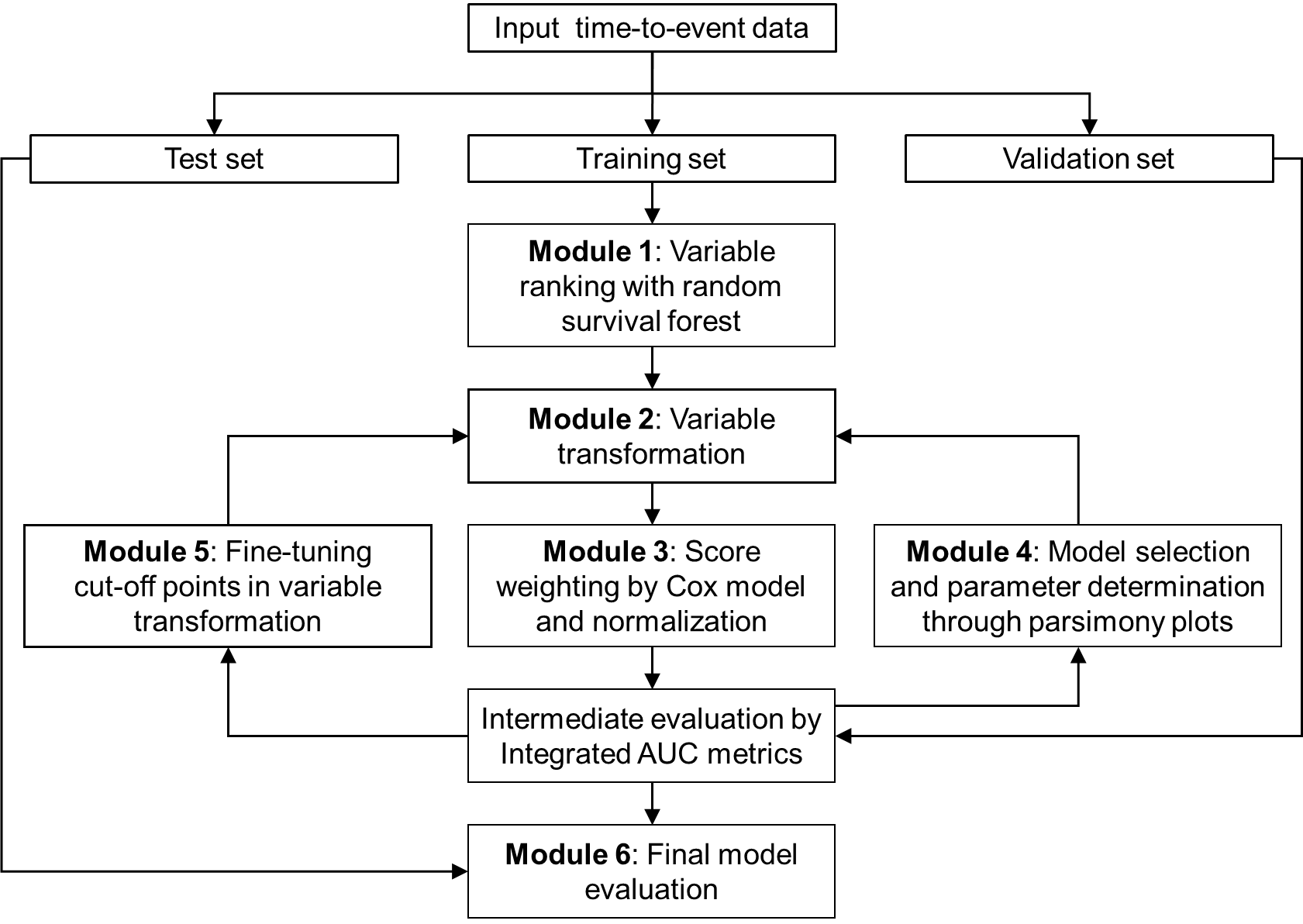


AutoScore-Survival consists of six modules: Module 1: variable ranking with random survival forest, Module 2: variable transformation or variable discretization, Module 3: score weighting by Cox regression and normalization, Module 4: model selection through parsimony plot, Module 5: fine-tuning cutoff points in variable transformation, and Module 6: final performance evaluation. The details are described in the publication (<https://doi.org/10.1016/j.jbi.2021.103959>). The proposed AutoScore-Survival framework can automatically generate a single indicative score for predicting patients' time-to-event outcomes. Users (clinicians or scientists) could effortlessly use our R package and demo codes to generate parsimonious time-to-event scores. We hope to see its application in various medical case studies.

Following the flow of the AutoScore-Survival, the training cohorts initially went through the algorithm, where the candidate variables were ranked by random survival forest in Module 1; continuous variables were discretized in Module 2; different categories were weighted by Cox regression in Module 3, and the candidate SERAP score was created.

Second, the number of variables was decided by the parsimony plot (i.e., model performance vs. complexity) (Module 4) on the validation cohort. The parsimonious models would be selected while maximizing predictive accuracy (integrated area under the curve).

Furthermore, the automatically generated cutoff values of each continuous variable can be fine-tuned by combining, rounding, and adjusting according to the standard clinical norm. (Module 5)

Lastly, we confirmed the variables and fine-tuned cutoffs. Then Modules 2 and 3 would be re-run to generate the final SERAP model. The performance final SERAP model was evaluated based on the integrated area under the curve and concordance index in Module 6.


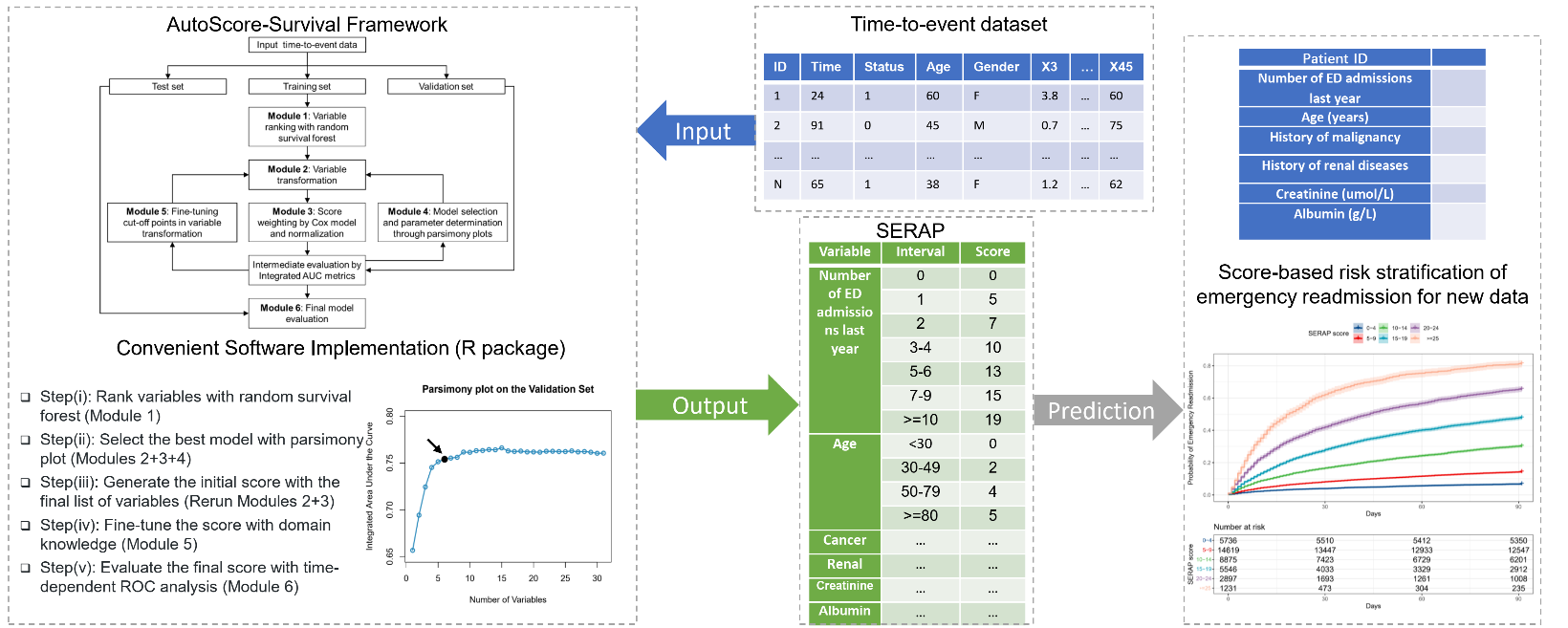
The graphical abstract of developing and validating the SERAP model through AutoSocre-Survival is described below. This is a summary of the whole process of model development and validation.
